# Challenges to informed choice counselling: a qualitative study of contraceptive self-care introduction in the Copperbelt Province of Zambia

**DOI:** 10.1136/bmjgh-2024-018764

**Published:** 2025-11-28

**Authors:** Jane Cover, Caitlin Corneliess, Monica Mutesa

**Affiliations:** 1 PATH, Seattle, Washington, USA; 2 MSI Reproductive Choices, London, UK; 3 PATH, Lusaka, Zambia

**Keywords:** Qualitative study, Health services research, Health education and promotion, Global Health, Delivery of Health Care

## Abstract

**Introduction:**

Contraceptive self-care adds a new dimension to informed choice counselling as providers communicate not only information about methods but also choice in how that method is to be administered. Even as many countries are scaling subcutaneous depot medroxyprogesterone acetate (DMPA-SC) for self-injection (SI), no studies have examined the potential repercussions for informed choice counselling. This qualitative study from Zambia investigates the content and quality of information given to family planning clients to understand whether DMPA-SC and SI are introduced in ways that respect informed choice.

**Methods:**

The study explored complex attitudes and practices through structured and in-depth interviews with 48 family planning clients and 24 providers from 24 public sector facilities in the Copperbelt Province of Zambia. Interviews were conducted in person, in a private setting, and in the preferred language of the participant. In-depth interviews were audio-recorded, translated and transcribed; the structured interview was entered electronically. Qualitative coding followed an inductive approach to identify key themes that resonate with the two groups of participants.

**Results:**

While providers do not appear to prefer specific methods, some express strong views about the appropriateness of different methods for adolescents, and these views may shape the tenor and content of their counselling sessions. The study found a lack of comprehensive, method-specific side effects information, which likely contributes to method discontinuation. While two clients were pressured to self-inject, the more common scenario was failure to discuss SI entirely or in sufficient detail for clients to make an informed choice. Providers expressed concerns around the type of client for whom SI is appropriate, showing reluctance to offer self-care options to adolescents and women with less education.

**Conclusion:**

Findings reveal the importance of reinforcing informed choice principles and monitoring the quality of contraceptive counselling when introducing new contraceptive methods and self-care innovations.

**WHAT IS ALREADY KNOWN ON THIS TOPIC**

- Even as contraceptive self-care is being introduced and scaled in many low- and middle-income countries, there is limited research focused on understanding when, how and to whom the self-care option is introduced in the context of family planning counselling.

**WHAT THIS STUDY ADDS**

- This study is the first to examine the nature of provider communication with women around self-care with a particular lens on the extent to which the information exchange reflects the principle of informed choice counselling.

**HOW THIS STUDY MIGHT AFFECT RESEARCH, PRACTICE OR POLICY**

- This study reveals that self-care complicates the family planning counselling session, necessitating efforts to monitor, measure and correct violations of informed choice that may arise in the context of contraceptive self-care introduction.

## Introduction

 The principle of informed choice is fundamental to rights-based family planning (FP) service delivery. This principle holds that clients select the method that best satisfies their personal, reproductive and health needs, based on a thorough understanding of their contraceptive options and free from coercion.^1^ New contraceptive product introductions may unintentionally drive biased counselling that violates informed choice, as introduction efforts organically focus on the new method. Progress in the scale-up of new methods is often tracked through method uptake data, and we have little information on whether new methods are offered in ways that are consistent with the informed choice principle. The shift towards rights-based FP that accelerated with the International Conference on Population and Development in 1994 has moved the field sharply away from method-specific targets. Yet recent studies have found violations of informed choice as providers feel urgency to advance a particular type of method, leading to coercion.^2 3^

Contraceptive self-care adds a new dimension to informed choice counselling, as providers must communicate not only information about methods but also offer a choice in how that method is to be administered. While self-injection (SI) of subcutaneous depot medroxyprogesterone acetate (DMPA-SC) can transform clients’ contraceptive access and autonomy by putting care directly into the hands of users, whether and how the self-care option is presented is critically important. Providers may exert pressure on women to engage in self-care, either in the interests of reducing their workload over time or out of genuine enthusiasm for enhanced reproductive autonomy for women. Conversely, providers may withhold information or otherwise interfere with effective self-care to save time in busy clinic settings or to maintain professional control and women’s dependency on their services.^4^

In 2018, the Zambian Ministry of Health began a progressive rollout of the new injectable method, DMPA-SC, including the option of SI. With this introduction, the ministry’s intent was to increase contraceptive prevalence (at 53% of married women) and to reduce unmet need (at 16%) by increasing the availability and diversity of contraceptive methods, building upon the popularity of injectable contraception (54% of the method mix).^5^ Most of the 3000+ providers trained in DMPA-SC and SI at the time of this study received training in 2019 using the innovative approach of leveraging supervision visits to provide on-the-job (OJT) training (in lieu of a formal cluster-based training approach). The training also included a refresher on basic FP knowledge and counselling.

Though informed choice counselling was not specifically emphasised in the OJT approach, it is part and parcel of preservice training for nurses and midwives, who customarily provide FP services in Zambia. The Ministry of Health of Zambia updated their Family Planning Counselling toolkit in 2021, encouraging health providers to engage with clients in a consultative process to ‘make informed decisions of their choice of contraception’.^6^ The counselling toolkit conforms to the GATHER counselling framework for FP: Greet, Ask, Tell, Help, Explain and Return.^7^ In Zambia, FP clinics customarily begin with group counselling where general FP information is provided. One-on-one counselling and method administration follow the group sessions.

The introduction of DMPA-SC for SI in Zambia creates an opportunity to examine the extent to which new methods and self-care options are introduced in the context of informed choice counselling. This qualitative study from the Copperbelt Province investigates the content and quality of information given to FP clients to understand whether DMPA-SC and SI are introduced in ways that respect informed choice. Understanding challenges in how information is communicated (or not) to FP clients is critical for guiding the introduction of new methods and self-care options.

## Methods

### Theoretical framework

Informed choice is a key element of quality of care (QOC). The well-established QOC framework put forth by Judith Bruce in 1990 articulates six dimensions, the first two of which are central to informed choice: choice of contraceptive methods and information given to clients.^8^ The Bruce QOC framework has withstood the test of time, laying the foundation for a 2020 QOC framework for self-care developed by the Self-Care Trailblazers Working Group.^9^ This framework sets standards for care across five dimensions, two of which pertain to informed choice: (1) information exchange and (2) interpersonal connection and choice. Within these dimensions, three standards reflect informed choice: clients can access comprehensible information that responds to their expressed needs and preferences and includes a range of options (standard 3.1); clients can access information regarding benefits, risks and side effects of a chosen self-care product prior to receipt (standard 3.2), and clients exercise choice, are not pressured or coerced, and give consent to use a self-care product (standard 4.4).

The QOC framework for self-care provides a useful architecture for the measurement of informed choice. Currently, however, standardised survey measurement approaches define and measure informed choice largely in terms of the Method Information Index (MII), which reflects the percentage of FP users who respond affirmatively to all of the following questions, indicating that they were: (1) informed of multiple methods of contraception that could be used, (2) informed about side effects or problems of the method used and (3) told what to do if they experienced side effects or problems.^10^ To these indicators, a fourth was recently added to create the MII plus (MII+): (4) told about the possibility of switching methods.^11^ This approach is limited in scope, focusing exclusively on information communicated and excluding both the voluntary choice component (ie, freedom from pressure) and the self-care dimension (ie, information and choice regarding the self-care option).

**Figure 1:**
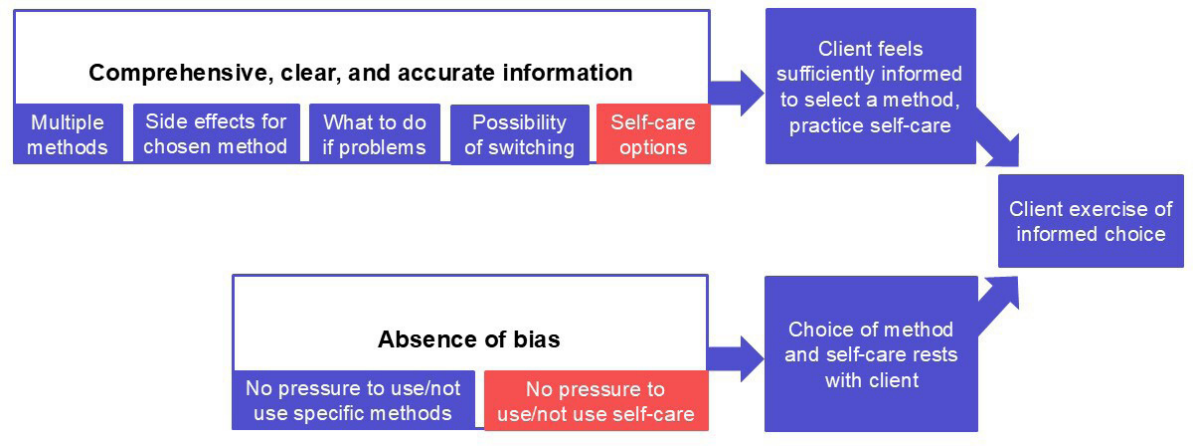
Conceptual framework for measuring informed choice.

Building upon the QOC framework for self-care and applying elements of the MII, we developed a framework to define and measure informed choice in a way that captures both choice and self-care (figure 1). The top half of the figure focuses on information provided on methods, side effects and their resolution, the possibility of switching and the self-care option, while the bottom half details the extent to which clients may experience method or self-care bias. Exercise of informed choice occurs when a client is sufficiently informed and able to choose without undue pressure. This framework guides our examination and interpretation of FP counselling practices in a context where DMPA-SC and SI have recently been introduced.

### Study design and objectives

The study was designed to uncover and explore learnings around informed choice counselling in the context of scaling up a new FP method (DMPA-SC) and SI as a mode of administration in Zambia. Specifically, this qualitative research study was conducted to (1) assess the extent and nature of any informed choice challenges with FP counselling, as experienced by clients; (2) identify provider attitudes and practices that may impact method choice or mode of delivery and (3) identify practical or operational challenges that may impact method choice or mode of delivery. The study approach was two-pronged, exploring complex attitudes and practices through structured surveys and in-depth interviews (IDIs) with both clients and FP providers. The study approach was informed by the standards for reporting qualitative research (SRQR) guidelines (see online supplemental material S1, SRQR guidelines).

### Sample and recruitment

The Copperbelt Province was selected for this study since the region was one of the first to roll out DMPA-SC for SI, and DMPA-SC uptake is relatively high. The Copperbelt contains the second and third largest cities in Zambia and is quite diverse with an agricultural and mining-based economy that attracts migrants from across Zambia. A total of 24 public facilities were purposively selected from two rural and six urban districts with the goal of maximising diversity of experiences with respect to FP service delivery. To be selected, a facility needed to have at least one FP provider trained in counselling of clients for DMPA-SC SI and have reported SI data in the last 12 months.

Client participants were women seeking FP services on the day the study team was visiting the facility, at least 18 years of age, able to communicate in English, Bemba or Nyanja (the principal languages of the Copperbelt region) and willing/able to provide informed consent. A total of 48 clients participated, two from each of the 24 facilities. Study team members approached clients at the end of their FP visit to request their participation and engage them in an informed consent process.

Provider participants were eligible if they were responsible for FP services at the facility on a consistent basis (a function that is usually assigned to nurses or midwives in Zambia), had received training in how to counsel women for SI and were willing/able to provide informed consent. A total of 24 providers participated, one from each of the 24 facilities. A sample size of 24 providers was expected to be sufficient to achieve saturation of themes, given general homogeneity among FP providers.^12^

### Data collection procedures

Interviewers were trained in research ethics and qualitative interviewing techniques, including techniques to build rapport and help participants to feel at ease. Interviews with FP clients were primarily conducted by female interviewers to enhance comfort with sensitive questions. The field team was entirely comprised of Zambians, and interview questions were reviewed by Zambian women of reproductive age, familiar with contraceptive service delivery in Zambian public facilities, to ensure questions were context-appropriate.

Structured surveys capture basic sociodemographic information about participants and enable quantification of simple metrics, allowing a more contextualised understanding of the in-depth interview findings. The structured survey for clients collected data on demographic characteristics; contraceptive history; discussion of methods, side effects and switching (MII+); SI information provided, awareness and interest; experience of counselling quality (Quality of Contraceptive Counselling scale)^13^ and the clinic environment on the day of her visit. With respect to providers, the structured survey collected data on demographic characteristics; FP methods normally provided at the facility and their availability; provider training experiences with DMPA-SC and SI; practices related to training clients for SI and attitudes towards SI.

IDIs delve into the experience of clients and providers and explore the rationales underlying behaviour. The client IDI guide included questions that explored the counselling experience, including information given on specific methods, their side effects and the SI option, and assessed their interest in SI. Clients were asked about perceptions of provider bias with respect to methods or self-care (see online supplemental material S2, IDI guides for specific questions asked). The IDI guide for providers included questions that explored method preferences, attitudes towards serving unmarried adolescent clients and experiences with offering SI training to clients (see online supplemental material S2, IDI guides).

All interviews were conducted in September 2022 and were in-person, in a location with auditory privacy at the clinic (in an office or in an outdoor seating area) and in the participant’s preferred language. Participants were reassured that their responses would not be shared with anyone outside of the research team, including clinic administrators or district supervisors. No individuals expressed concerns about confidentiality.

### Data analysis

The general analysis approach was to triangulate findings from providers and those of clients to understand and compare behaviours and perspectives.

Structured interview data was entered electronically using Open Data Kit (ODK). Responses to quantitative questions were analysed using STATA software. Because of the small sample size, tests of statistical significance were not performed.

In-depth interviews were audio-recorded, translated and transcribed verbatim. For each research assistant, the first interview was evaluated for quality and feedback was provided before continuing interviews. Qualitative analysis employed the Atlas.ti software, using an inductive coding approach to identify key themes that resonate across groups of participants. Code interpretation was assessed by multiple authors, and discrepancies were resolved.

For the quantitative data, all questions were answered for all participants. For the qualitative data, saturation in themes was achieved as evidenced by diminished new information. Since data analysis was initiated sequentially after data collection was completed, the full proposed sample (of 24 providers and 48 clients) was interviewed.

## Results

### Client and provider characteristics

The average participating FP client was a 27-year-old married woman with at least some secondary education. Over one-third were first-time method users, while the remaining two-thirds were continuing the same method or switching to a new method. About two-thirds received an injectable and four were self-injecting.

The average participating provider was a 38-year-old nurse or midwife who had worked in FP for 6 years. While many had received contraceptive counselling training, fewer had received training focused on adolescent reproductive health. All but two providers were trained in DMPA-SC SI, most often through informal training by a colleague or supervisor. Most were satisfied with the training received and confident in their ability to train clients. Although four in five had trained clients to self-inject at some point, the median number trained in the past month was two clients, with nearly half having trained no clients (see online supplemental material S3 for a table of participant characteristics).

Analysis of findings with respect to the comprehensiveness of information provided on methods, side effects, switching and self-care (the upper half of figure 1) reveals challenges centred on limited discussion of alternative methods, particularly with new clients, failure to comprehensively discuss method side effects and failure to train clients on the SI option, despite considerable interest.

### Comprehensive method information

When providers were queried in the IDIs about the information they provide about methods, their responses suggest familiarity with the principle of informed choice. However, from the client’s perspective, communication about methods during their counselling sessions was not always comprehensive. From the survey data (table 1), while four in five clients learnt about at least one method other than the one provided, fewer first-time method users were told of methods other than the one they were given. When methods were discussed, the focus tended to be on short-term methods (particularly injectables and oral contraceptives).

**Table 1:** Discussion of methods during FP counselling (client structured survey)

	FP clients (n=48)
Whether methods discussed, other than the one provided (today or past visit)	79.2%
Switchers (n=9)	100%
Continuers (n=22)	81.8%
New users (n=17)	64.7%
Mean number of methods discussed (if any discussed)	4
Methods discussed (if any method discussed)	n=25
Injectables (intramuscular or subcutaneous)	92%
Oral contraceptives	80%
Implants	64%
Condoms	56%
Intrauterine device	48%

FP, family planning.

A key theme that emerged from client IDIs was a tendency for providers to discuss in a general way the benefits of FP in lieu of a comprehensive discussion of method options, as the quote below from a new user illustrates.

They told us how to care for our children and that we should get family planning pills. We shouldn’t have a child who is maybe six months old and then have another one, but maybe after five or four years that’s when you could have another child. I wasn’t told about the different types of family planning methods. DMPA-SC (new user), 20s.

### Comprehensive side effects information

**Figure 2:**
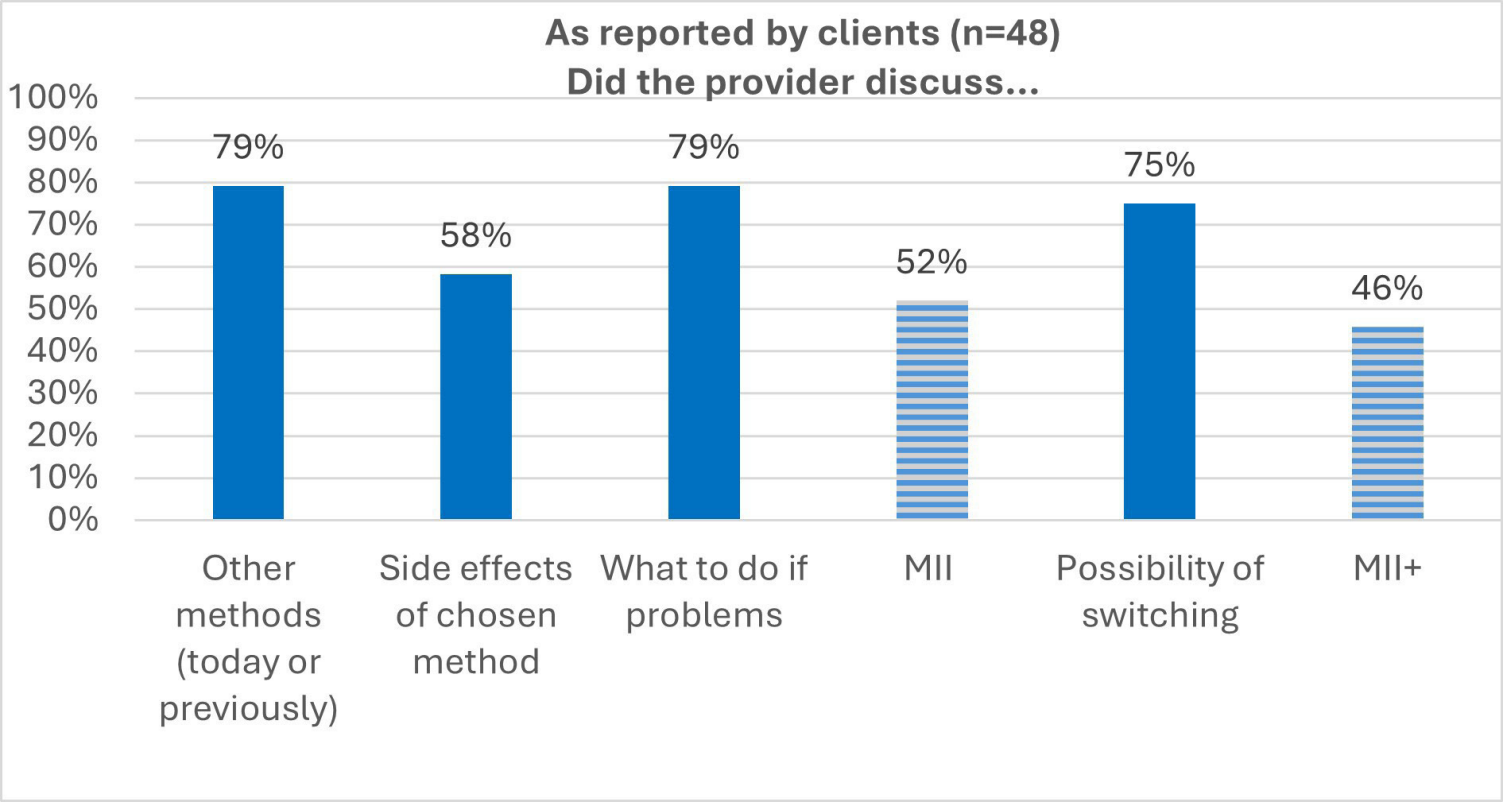
Method Information Index (MII) and MII plus.

Inconsistent discussion of side effects contributed to a low MII score, with only about half of providers discussing all elements of the index, whether MII or MII+ (figure 2).

The IDIs reveal that, while most clients knew to return in the event of side effects, many did not know which side effects were possible for their method. This lack of method-specific side effects information was a major theme that emerged. One client discussed potential side effects she has experienced while lamenting the lack of education by health providers during FP visits. Another client’s comment demonstrates that when there is an information void on side effects, clients fill in using information from their networks.

There are no side effects with this method. No, we were not told anything of the sort. Now that you mentioned them, sometimes I experience headaches, dizziness, and start seeing black things on my face, but I have never been told about it here. When we reach here, the nurse does not say much, no learning happens and only a few words are said, then we are later just injected and off we go. DMPA-SC (switching user), 20s.

The provider never mentioned any side effects but from what I have heard in my community there are some whose bodies fail to adapt to the contraceptive, which leads to having periods throughout without stopping. Others slim down and for others it doesn’t react negatively in any way. DMPA-SC (new user), <20s.

### Method switching information

The survey results suggest most clients were told of the option to switch methods, and many of those who were not told explicitly indicated they felt free to change methods. However, a small number of clients (n=6) were specifically discouraged from switching methods.

The only thing she mentioned is that if I keep switching contraceptives, I may encounter problems.DMPA-IM (continuing user), <20s.

### SI information

Turning next to the question of information given to clients about self-care, providers reported that discussion of SI is a routine part of FP counselling. However, contrary to provider claims, discussion of SI during counselling is not routine, except for among DMPA-SC clients. Just over half of clients reported that a provider had ever told them about the SI option, with much higher rates among clients using DMPA-SC relative to DMPA-IM users (table 2). Despite the lack of information, there is substantial interest in SI overall, not only among DMPA-SC users but also among DMPA-IM users and even among women using other methods.

**Table 2:** Discussion of, and interest in, self-injection (client structured survey)

	Family planning clients (n=48)
Told about self-injection by a provider, today or previously	54.2%
DMPA-SC client (n=14)	78.6%
DMPA-IM client (n=16)	43.8%
Client using non-injectable method (n=18)	44.5%
Heard of self-injection before today’s visit	60.4%
Self-injection experience (among DMPA-SC clients)	(n=14)
Self-injected today (only)	3
Self-injected today and previously	1
Has never self-injected	10
Would consider self-injecting	62.5%
DMPA-SC clients (n=14)	85.7%
DMPA-IM clients (n=16)	56.3%
Clients using non-injectable methods (n=18)	50%
High interest in self-injection* (I am already self-injecting or very interested in trying self-injection.)	58.3%
DMPA-SC clients (n=14)	71.4%
DMPA-IM clients (n=16)	56.3%
Clients using non-injectable methods (n=18)	50%
No interest in self-injection at all* (self-injection is just not for me.)	
Injectable clients (SC or IM) (n=30)	26.7%
Clients using no-injectable methods (n=18)	44.4%

DMPA-IM, intramuscular depot medroxyprogesterone acetate; DMPA-SC, subcutaneous depot medroxyprogesterone acetate; IM, intramuscular; SC, subcutaneous.

The IDIs reveal that client’s views on SI are shaped by their lack of information and training, and we find a common perception that SI is beyond their capacity, almost inconceivable.

What I know is that I am able to make mistakes when self-injecting. Sometimes I may not take the dosage in the correct way. I think it’s way different if it’s administered by a provider. I have not much information because the provider did not explain much on the self-injected contraceptive. DMPA-IM (continuing user), <20s

Well for me, I have never been taught about self-injection. The nurse tells us that you do not have to get the injection and inject yourself because you do not have experience with it and you have never learned about it, so you cannot inject yourself and bring problems to yourself. Sometimes she teaches those who are interested, but she refuses us to self-inject, telling us that every time we need an injection, we just have to come and get it from here. DMPA-IM (continuing user), 20s

Though only 4 of the 48 clients had self-injected previously, clients indicated that, if training were provided, they would consider SI.

According to how she explained and showed me, I am willing to self-inject. If you are told what to do, you can self-inject. DMPA-SC (new user), <20s.

With regard to method and self-care bias—the extent to which clients may feel pressured to choose particular methods or modes of administration (the bottom half of figure 1)—analysis shows that providers have diverse perspectives on methods appropriate for adolescents, and a large minority of providers believe contraceptive injections should only be given by a provider. In terms of the impact of bias of contraceptive method and self-care choice, most clients felt no pressure to select a certain method or to self-inject DMPA-SC. However, two clients expressed that they felt pressured by providers to self-inject.

### Presence or absence of method bias

When providers were asked if they advance some methods over others, a relatively common response (n=8) was that clients are free to choose.

On that one, it is hard to speak on their behalf. As a provider, all I need to do is educate them and then it is their choice to make. Nurse, offering FP for 1 year, 30s.

However, about half of providers (n=12) indicated that some methods are inherently better than others, with all but two preferring long-term methods.

The most suitable ones are the long-term methods where a woman will come and access the method and go, coming back after a long time… Long term methods are suitable because they give a woman time to do other things. In-charge, offering FP for 18 years, 40s.

With respect to methods suitable for adolescents, providers showed more pronounced method bias and greater diversity of method preferences. Many proposed condoms and long-term methods as ‘best’ for adolescents.

When giving a service to adolescents, we are looking at the future, what will happen to them. We understand that this time around, they want to explore and enjoy, but tomorrow, they will want to conceive. What will happen if they fail. Therefore, it is better to encourage them to use a condom. Nurse, 6 years offering FP, 40s.

What makes the long-term method better for the adolescent is because the adolescent will have time, even to study if she is at school, and its less inconveniencing. Because these short-term methods, she needs to be coming to the facility… so usually maybe she is busy and because of stigma she can get maybe the first jab and then after three months she doesn’t come (back). In-charge, offering FP for 18 years, 40s.

However, providers were of diverse minds with respect to appropriate methods for adolescents, and some appeared to speculate with respect to the adolescent client’s duration of use when considering what methods to offer adolescents.

I think implants are not okay because adolescents are still young and others might get married, and those long-term ones, we do not encourage removing before the expire data because we avoid wasting stock. Nurse, offering FP for 4 years, 30s.

Not only did more providers prefer specific methods outright, but only one provider indicated that the best method for an adolescent is the one she wants to use.

### Presence or absence of self-care bias

**Figure 3:**
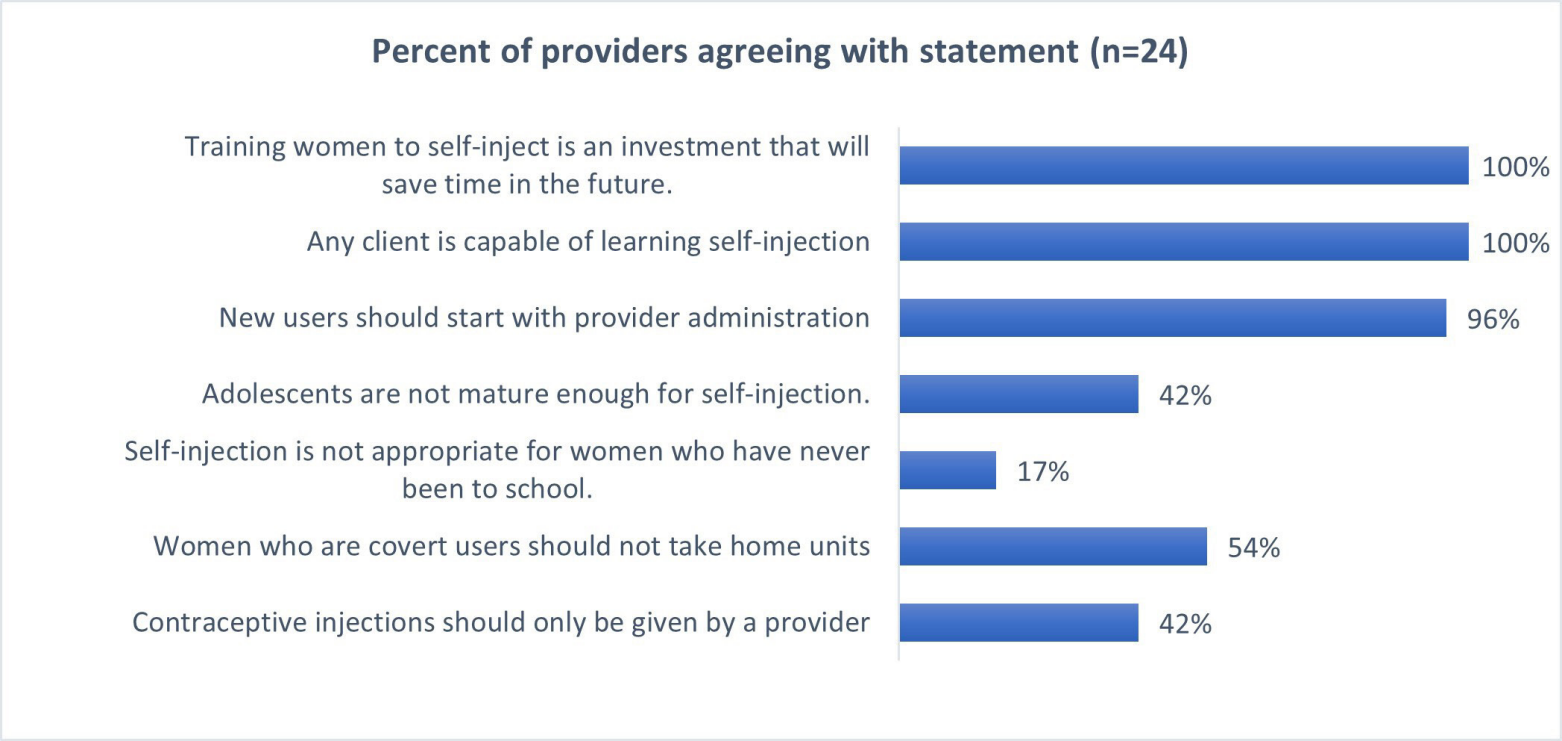
Provider views on self-injection.

When asked their views on SI in the structured survey, providers expressed mixed opinions. On the one hand, all providers queried felt that any woman could learn SI and that SI is an investment that would save time in the long run (figure 3). However, many have strong views about who should self-inject, with nearly all believing new injectable users should not self-inject and a substantial minority believing that adolescents are not mature enough for SI. Fundamentally, a large minority believes contraceptive injections should only be given by a provider.

In IDIs, some providers identified a specific type of woman—those who are busy, educated and working—as good candidates for SI, with some expressing concern about non-literate clients’ and adolescents’ ability to manage self-care.

We believe it is not everyone that went to school that is knowledgeable enough and able to grasp a skill fast. For those that are not educated, we haven’t yet given one because maybe if we give them, they won’t be able to store it okay and maybe when injecting themselves, they won’t follow the aseptic techniques. Nurse, offering FP for 4 years, 30s.

For the adolescent, we haven’t yet started (with SI) because that age is very, I can say experimental. I can give her then instead of her using it on herself, she’ll go and use it on a friend, maybe she is not even at the age of menstruation… For the adolescent, it’s no. Nurse, offering FP for 4 years, 30s.

However, when asked directly about serving adolescents, just two providers opined that adolescents should be actively discouraged from self-injecting.

A substantial share of providers insisted that there is no ‘type of person’ for SI, and the choice to self-inject rests with the client.

While no providers expressed outright opposition to SI in the IDIs, when asked about challenges, comments reveal their reservations.

Women do not follow time when it comes to inject themselves. They inject at any time, which may lead to forgetting. When they are coming to the facility, they follow time, knowing that the nurse will not tolerate that. Nurse, offering FP for 6 months, 50s.

### Impact of bias on choices

Turning to any impact of method bias on client choice, most clients (n=25) indicated they felt free to choose the method they wanted and did not feel coerced in any way by the provider. For the small minority (n=3) who perceived that the provider wanted them to use a particular method, clients insisted on their preferred methods.

The provider had a different method in mind – the implant – but I wanted the injection. I decided to use Depo. DMPA-IM (continuing user), 20s.

Turning next to the perception of clients as to whether they feel pressure to self-inject, nearly all indicated that there was no pressure to self-inject and that they were able to receive DMPA-SC, if desired, via provider administration.

They do not force. They ask you what you want, if you would manage to self-inject, they teach you. If you refuse, they inject you themselves. –Oral Contraceptives (continuing user), 30s.

However, two clients (among the four self-injectors) spoke of pressure from providers to self-inject.

They showed me how to inject myself. It (the information) was 100% clear, only that it was a challenge for me. I was not comfortable and prepared. I was even shivering, and my hands were shaking. However, I had to do it. For me, self-injection is not working well. The injection is good but if they remove self-injection, it will be better. I do not want to do self-injection. Self-injector (switching, first time self-injecting), 20s.

That’s what I was taught, that ‘no, you just have to do it at home. What we have taught you here is what you are going to do at home.’ It wasn’t like, ‘since you don’t feel like injecting yourself you can come back.’ That wasn’t how I was taught. It wasn’t my free will to say that I’ll come (back), I can’t inject myself. They just told me to do it at home and then I come to collect another one. So, I can say yes to this question [about pressure to self-inject] because it wasn’t my will. They should have let me have the option to say ‘no, I’ll continue coming so you can inject me.’ Self-injector (continuing user, first time self-injecting), 30s.

## Discussion

This study illuminates the FP counselling session in the context of new self-care method introduction, drawing upon client and provider perspectives to understand the nature and quality of counselling, made more challenging by the added dimension of self-care. This is the first study to assess threats to informed choice counselling presented by the added self-care dimension and responds to a call for more research in this area.^14^ While our results focus on the SI of DMPA-SC in Zambia, there are broader implications for self-care initiatives. We propose a person-centred approach (figure 1) that extends the principle of informed choice to encompass not only choice of method but also choice of mode of administration and applied our conceptual framework to assess FP counselling quality in Zambia.

This study did not find pronounced challenges related to a lack of information about methods or strong method bias. Importantly, however, new users may have more constrained method selection, given the sizeable share who reported that no other methods than the one given were discussed.

While providers do not appear to be preferring specific methods overall, some express strong views about adolescent use of specific methods, and these views shape the tenor and content of their counselling sessions. It has frequently been observed that providers’ unfounded concerns over the fertility impacts of hormonal and/or long-acting methods may result in age and parity restrictions.^15^ It is less well-established that providers may dissuade adolescents from using implants because they are on the cusp of their childbearing years and therefore would potentially ‘waste’ a long-acting method. In addition to limiting choice, this misplaced focus on using a method for its full duration of efficacy may translate into restrictions placed on method switching. This finding bears further scrutiny to better understand how pervasive this form of method bias may be. More generally, with respect to adolescent clients, there is an ongoing need to monitor provider adherence to informed choice for method selection. This observation echoes findings shown across a wide range of settings documenting limited method selection affecting adolescents, including in Nigeria, where providers may limit provision of DMPA-SC for unmarried adolescents, despite high levels of stated intention to provide it.^16^

Equally concerning as method bias is the lack of comprehensive, method-specific side effects information, which drove down the MII and MII+ indices. Failing to systematically discuss side effects (anticipatory counselling) encourages rumours and misconceptions, contributing to high rates of method discontinuation.^17^

With respect to self-care, two clients discussed feeling pressured to self-inject. These findings are concerning and emphasise the need for health worker guidance on the right to receive provider-administered injections, if that is the client’s preference. Fortunately, these cases do not appear to be the norm, and the much more common scenario was either omission of SI altogether or failing to provide comprehensive information for clients to make an informed choice. Only about half of clients reported that a provider had *ever* told them about the SI option, and their qualitative interview responses reflect how foreign the concept of SI remains for them, fully 4 years after the roll-out of SI services. In addition, we saw provider concerns around the type of client for whom SI is appropriate, with particular reticence around SI services for adolescents and women with less education. These results are consistent with provider concerns shared in other countries,^18 19^ despite evidence that both groups are capable of self-injecting safely and effectively.^20 21^

A meta-analysis of randomised control trials shows self-administration of DMPA-SC increased continuation compared with provider administration in Malawi and the USA.^22^ Contrasting these results with our study, when the SI option is not presented to clients, or when clients are not adequately counselled on side effects, the potential for improved continuation rates, and thus reduction of unintended pregnancies may be diminished. Where the author’s analysis shows the opportunities for improved continuation rates with DMPA-SC SI demonstrated in research settings, our study in Zambia documents real-world experiences with scaling up this new method and mode of provision in public sector non-research settings. Low MII/MII+ scores and low client knowledge of SI indicate that full counselling in this setting was less successful.

These findings suggest the need for guidance and supportive supervision focused on when and how to talk about SI, reinforcing for providers that self-care can be practised safely and effectively and that all women, regardless of education or age, have a right to practise self-care. The introduction of new methods and self-care practices presents an opportunity for health system strengthening, and accordingly, provider training should involve values clarification, address misconceptions and include a refresher on informed choice counselling for all methods and modes of administration. Lengthier programme monitoring may be required to more fully document results of scaling up self-care options within health systems, as noted by a recent scoping review of self-administration of DMPA-SC.^23^

A person-centred approach to self-care is the first principle named in the WHO self-care guidelines. Specifically, a person-centred approach ‘sees individuals as active participants in, as well as beneficiaries of, trusted health systems that respond to their needs, rights and preferences in humane and holistic ways’.^24^ Consistent with a person-centred approach, the decision to practise self-care should be founded upon comprehensive information about self-care options and the freedom to choose them (or not) without undue influence. Rather than evaluating the success of new contraceptive products or self-care introductions primarily in terms of uptake,^25 26^ we suggest refining the metrics by which we measure success, incorporating elements of counselling quality to better align with broader goals around choice and autonomy.

## Strengths and limitations

As with most social science research, social desirability bias, in which participants present themselves in ways they believe will be viewed favourably by others, may have influenced responses. The study was designed to minimise social desirability bias; however, with clients asked about provider behaviour, rather than relying (exclusively) on provider self-reports. While there remains a risk that providers would adjust their counselling practices, given the knowledge that clients will be interviewed, lines of inquiry with clients were not revealed to providers. Each provider was recruited, consented and interviewed after both client interviews were completed.

Conducting interviews at the facility may have influenced responses, as participants may not have felt as free to disclose their views as they would, perhaps, in another setting. That said, the team reassured participants of the confidentiality of their responses and conducted interviews in private to encourage forthright responses.

Finally, the study focused on the experience of women and providers in a single province, and it is not known the extent to which their experiences were typical for women receiving public sector FP services in Zambia more generally.

## Conclusion

Contraceptive and self-care introduction efforts must be sensitive to the potential for bias—either for or against the new method or mode of administration. Provider training should ensure competence in new method provision as well as consistency in offering new options. Efforts to measure, monitor and correct bias should be fundamental elements of initiatives to introduce and scale new methods and practices.

## Supplementary material

10.1136/bmjgh-2024-018764 online supplemental file 1

10.1136/bmjgh-2024-018764 online supplemental file 2

10.1136/bmjgh-2024-018764 online supplemental file 3

10.1136/bmjgh-2024-018764 online supplemental file 4

## Data Availability

Data are available upon reasonable request.
