# Supplementary material for: Challenges to informed choice counselling: a qualitative study of contraceptive self-care introduction in the Copperbelt Province of Zambia
Source: BMJ Glob Health. 2025 Nov 28;10(Suppl 6):e018764. doi: 10.1136/bmjgh-2024-018764 (PMC12673566; doi:10.1136/bmjgh-2024-018764)
Supplement: online supplemental file 2 [file bmjgh-10-Suppl_6-s002.docx]

**Supplemental materials: IDI Interview Guides**

**CLIENT IN-DEPTH INTERVIEW GUIDE**

**[BEFORE BEGINNING THESE QUESTIONS, TURN ON THE AUDIO RECORDER, TEST THE SOUND, AND SAY THE DATE, TIME, AND THE PARTICIPANT ID NUMBER.]**

| **Date** | DD/MM/YYYY ………… **\|__\|__\| \|__\|__\| \|__\|__\|__\|__\|** |
| --- | --- |
| **District** |  |
| **Facility Name** | ………………………………………………….. |
| **Interviewer ID** | ………………………………………………….. |
| **Participant ID** | ………………………………… |
| **Start time** | HH/MM/SS ………………... **\|__\|__\| \|__\|__\| \|__\|__\|** |
| **Audio recording ID** |  |

**I would like to discuss in a little more detail what occurred during your family planning visit today.** **As I mentioned earlier, I have turned on the audio recorder so I don’t miss anything that you say.**

1. During your visit today, what were you told about the different FP methods?

**Probe**: What advantages or disadvantages did they mention of specific methods?

**Probe**: What else did you learn about different FP methods?

**Probe***:* How prepared did you feel to choose FP method?

1. Sometimes providers have preferences for certain types of methods. Did you feel that the provider had a particular method they wanted you to use? Which method was that? Why do you think the provider wanted you to use that method? Did you feel completely free to refuse that method or did you feel pressure to accept it?
2. Were there any methods that the provider discouraged you from using? If so, which method(s) did the provider discourage you from using? Why did the provider say that method(s) would not be good for you?
3. What possible side effects or problems with your method did the provider mention? What were you told to do if you experienced side effects?
4. How free do you feel to switch to another method if the one you are using doesn’t work well for you? Did the provider specifically tell you that you can switch methods later?
5. What are some of the challenges that you experience when you come to the clinic for family planning? Tell me about any frustrations you have experienced, either at this visit, or in the past.
6. What about your interactions with the providers? What have they been like?

**Probe**: How well do they treat you here?

**Probe**: Have you ever felt judged or disrespected by the providers at this facility?

**I want to learn more about what you think of Sayana Press and self-injection.**

1. Some providers consider Sayana Press to be a method that is only for women who are willing to self-inject. Have you ever been told that if you want to use Sayana Press, you must learn to do self-injection? Have you ever felt pressured by a provider to take up self-injection?
2. How much, if anything, have you learned from providers about self-injection? How was self-injection presented to you by the provider? What is your understanding of the benefits of self-injecting?
3. How seriously have you considered the option of self-injection? What if anything about self-injection is appealing or unappealing to you?

**IF NOT CURRENTLY SELF-INJECTING: END INTERVIEW**

**IF CURRENTLY SELF-INJECTING:**

1. What was your self-injection training like? Describe it for me.

**Probe**: Were you trained in a group or individually?

**Probe**: How clear and complete was the information you were given?

**Probe**: How comfortable and prepared did you feel to self-inject at the end of training?

1. Tell me more about your experience with self-injection since your training. How is self-injection working out for you?

**Probe**: What has gone well and what has not gone so well?

**Probe**: What three words would you use to describe your self-injection experience?

***We thank you very much for your time and your contribution to this study.***

***PROVIDER IN-DEPTH INTERVIEW GUIDE***

**[BEFORE BEGINNING THESE QUESTIONS, TURN ON THE AUDIO RECORDER, SAY THE DATE, TIME, AND THE PARTICIPANT ID NUMBER.]**

| **DATE** | DD/MM/YYYY ………… **\|__\|__\| \|__\|__\| \|__\|__\|__\|__\|** |
| --- | --- |
| **District** |  |
| **Facility Name** | ………………………………………………….. |
| **Interviewer ID** | ………………………………………………….. |
| **Participant ID** | ………………………………… |
| **Start time** | HH/MM/SS ………………... **\|__\|__\| \|__\|__\| \|__\|__\|** |
| **Audio recording ID** |  |

**Introduction:**

As I mentioned earlier, I am using the audio recorder so I don’t miss anything that you say. Please keep in mind that your answers are completely confidential and will not be shared with anyone from this facility, the district, or the ministry of health, and will not be associated with you by name in any publication or report.

1. I’d like to learn about what it is like to provide family planning at this facility. What does a typical day look like for you?

**Probe**: Walk me though your workday from start to finish.

1. Which FP methods are most popular with women? Which methods do you think are more suitable or ideal for women to use?

**Probe**: What about newly introduced methods, like Sayana Press or the longterm methods that are more effective? Are those methods better and deserving of extra promotion by providers?

1. Are there particular methods that you do not usually counsel clients about unless they ask specifically for them? Which ones and why?
2. How is your approach to counseling different when you have an adolescent client? Under what conditions would you be reluctant to offer contraceptive methods to adolescents?

**Probe:** For example, if she is unmarried? Or young, say 16 years old? Or is using without her parent’s knowledge?

1. Which methods do you think are more, or less, appropriate for adolescents? What makes those methods better or worse for adolescents?

***Next I’d like to ask some questions about Sayana Press self-injection.***

Remind me again, did you say that you have trained any women in how to self-inject DMPA-SC/Sayana Press?

**[IF NO]**

1. What are some of the reasons why you have not trained any women in how to self-inject?
2. What, if anything, would make it easier for you to offer self-injection training?

[END INTERVIEW]

***[IF YES]***

1. If a client doesn’t ask you about it, how do you decide whether to mention the option of self-injection to her?

**Probe**: What factors influence your decision of whether to offer to train a woman to self-inject?

1. How much does your decision of whether to discuss self-injection with a client depend on the type of person she is, and whether you think she will be successful?

**Probe**: Would you discuss self-injection with covert users who are using FP without the partner’s knowledge? What about adolescents? What about women who have never been to school?

1. What happens with self-injection training if you are running low on Sayana Press units?

**Probe**: Do you stop training women to self-inject until you receive more Sayana Press or do you train them anyway? Do you still give out units to take home when you are low on stock?

1. What happens if a woman asks to use Sayana Press, but she is not interested in learning self-injection? What would you normally tell her?
2. Thinking about your experience with training clients to self-inject, what do you find most difficult about it?

**Probe**: What challenges have you faced in offering Sayana Press for self-injection? **Probe**: What solutions have you found?

1. With your experience with self-injection so far, have you observed any benefits for the clinic? Do you think in the future you will see benefits? In what way?
2. The MOH and some NGOs have put a lot of emphasis on self-injection and Sayana Press. To what extent do you feel pressured to promote self-injection to clients by facility managers, district supervisors, or NGOs?
