# Supplementary material for: Challenges to informed choice counselling: a qualitative study of contraceptive self-care introduction in the Copperbelt Province of Zambia
Source: BMJ Glob Health. 2025 Nov 28;10(Suppl 6):e018764. doi: 10.1136/bmjgh-2024-018764 (PMC12673566; doi:10.1136/bmjgh-2024-018764)
Supplement: online supplemental file 3 [file bmjgh-10-Suppl_6-s003.docx]

**Supplemental materials: Profile of study participants**

**(client and provider structured surveys)**

| **FP client demographics and contraceptive use** | **FP clients**  **(n=48)** |
| --- | --- |
| Mean age (range) | 26.8 (18-52) |
| Mean years of education (range) | 8.9 (0-15) |
| Married (n) | 72.9% (35) |
| Contraceptive experience (n)  New users  Continuing same method  Switching to a new method | 35.4% (17)  45.8% (22)  18.8% (9) |
| Method adopted, percent using (n)  DMPA-IM  DMPA-SC  Implant  IUD  Oral contraceptives  Emergency contraception | 33.3% (16)  29.2% (14)  25.0% (12)  2.1% (1)  8.3% (4)  2.1% (1) |
| Currently self-injecting (among injectable users) | 13.3% (4) |
| **FP provider demographics and training experience** | **FP providers**  **(N=24)** |
| Female | 96% (23) |
| Mean age of providers (range) | 38 (28-63) |
| Job cadre  Facility in-charge  Nurse, Midwife or Nurse/midwife | 20.8% (5)  79.2% (19) |
| Mean tenure working in family planning | 6.4 (7 mos. – 18 yrs.) |
| Perception of workload  Overloaded  Reasonable  Under-booked | 33.3% (8)  62.5% (15)  4.2% (1) |
| Ever received in-service training in FP counseling | 62.5% (15) |
| Ever received in-service training in adolescent reproductive health | 46% (11) |
| Training in DMPA-SC self-injection  Formal training by an external trainer  Informal training by a colleague or supervisor  Not trained | 91.7% (22)  31.8% (7)  62.5% (15)  9.1% (2) |
| Satisfied with self-care training (of those who were trained, n=22) | 86% |
| Confident in ability to train clients (all providers) | 91% (22) |
| Ever trained clients to self-inject | 83.3% (20) |
| Number trained past 30 days (median)  0  1–10  11–25 | 2  45.8% (11)  29.2% (7)  25.0% (6) |
