# Supplementary material for: Challenges to informed choice counselling: a qualitative study of contraceptive self-care introduction in the Copperbelt Province of Zambia
Source: BMJ Glob Health. 2025 Nov 28;10(Suppl 6):e018764. doi: 10.1136/bmjgh-2024-018764 (PMC12673566; doi:10.1136/bmjgh-2024-018764)
Supplement: online supplemental file 4 [file bmjgh-10-Suppl_6-s004.docx]

**Supplemental Materials**

**Reflexivity statement**

1. **How does this study address local research and policy priorities?**

This study was designed to support the Ministry of Health in Zambia with their efforts to scale up DMPA-SC for self-injection, and findings have been used for supportive supervision to improve service delivery.

1. **How were local researchers involved in study design?**

This was a small study with a small team in Zambia and collectively, we shaped the study design, the approach to field work, the tools employed, as well as discussion and interpretation of the findings. The field research team were all Zambians.

1. **How has funding been used to support the local research team?**

Funding was used to support the training of the study team in research methods, field visits, as well as the dissemination of results in-country and the preparation of this paper for publication.

1. **How are research staff who conducted data collection acknowledged?**

Research staff are acknowledged through an acknowledgement statement, as follows: The authors wish to acknowledge the support staff and research assistants who made this study possible, including Thandiwe Tsibulinjase, Joel Ngulimba, Joseph Mwanza, Liness Shasha, and Iwell Mwanza. We also thank the women and family planning providers of Copperbelt Province who were so generous with their time in sharing their experiences.

1. **Do all members of the research partnership have access to study data?**

All members of the team have access to the data.

1. **How was data used to develop analytical skills within the partnership?**

The data was explained and discussed with the data collection team, building their capacity for data interpretation and analysis.

1. **How have research partners collaborated in interpreting study data?**

During the study launch, the co-PIs collaborated with the data collection team to review, discuss, and interpret preliminary findings. The three authors all contributed to interpreting the findings.

1. **How were research partners supported to develop writing skills?**

MM, CC, and JC all contributed to the drafting of the manuscript.

1. **How will research products be shared to address local needs?**

The findings from this study have been shared locally.

1. **How is the leadership, contribution and ownership of this work by LMIC researchers recognised within the authorship?**

One of three co-authors (MM) is Zambian and her contributions are noted in the contribution statement.

Contribution statement: JC and MM served as co-principal investigators, designing the study, the approach, and the instruments. MM oversaw field work. JC conducted the analysis; JC, CC, and MM collectively discussed and interpreted key themes that emerged. JC, CC, and MM all contributed to writing the paper.

1. **How have early career researchers across the partnership been included within the authorship team?**

We did not have any early career researchers on the authorship team.

1. **How has gender balance been addressed within the authorship?**

All three authors are female.

1. **How has the project contributed to training of LMIC researchers?**

The field team is comprised of 4 Zambians, early in their careers, who participated in a multi-day training in research methods, informed consent processes, interviewing techniques, etc. The co-investigator is herself Zambian.

1. **How has the project contributed to improvements in local infrastructure?**

This project has not directly contributed to improvements in local infrastructure.

1. **What safeguarding procedures were used to protect local study participants and researchers?**

As per the informed consent procedures, local study participants were informed of their rights to decline to participate, to decline to answer any questions without explanation, and to withdraw from the study at any time. Strict confidentiality of research findings was enforced. Study teams were supported by the co-investigator, who was in the field with them during data collection to address any unforeseen events (there were none).
